# Dwarfism with joint laxity in Friesian horses is associated with a splice site mutation in *B4GALT7*

**DOI:** 10.1186/s12864-016-3186-0

**Published:** 2016-10-28

**Authors:** Peter A. Leegwater, Manon Vos-Loohuis, Bart J. Ducro, Iris J. Boegheim, Frank G. van Steenbeek, Isaac J. Nijman, Glen R. Monroe, John W. M. Bastiaansen, Bert W. Dibbits, Leanne H. van de Goor, Ids Hellinga, Willem Back, Anouk Schurink

**Affiliations:** 1 Department of Clinical Sciences of Companion Animals, Faculty of Veterinary Medicine, Utrecht University, PO Box 80154, NL-3508 TD Utrecht, The Netherlands; 2 Animal Breeding and Genomics Centre, Wageningen University, PO Box 338, NL-6700 AH Wageningen, The Netherlands; 3 Department of Equine Sciences, Faculty of Veterinary Medicine, Utrecht University, Yalelaan 112-114, NL-3584 CM Utrecht, The Netherlands; 4 Department of Medical Genetics, University Medical Center Utrecht, PO Box 85090, NL-3508 AB Utrecht, The Netherlands; 5 Dr. van Haeringen Laboratorium B.V., PO Box 408, NL-6700 AK Wageningen, The Netherlands; 6 Koninklijke Vereniging “het Friesch Paarden-Stamboek”, PO Box 624, NL-9200 AP Drachten, The Netherlands; 7 Department of Surgery and Anaesthesiology of Domestic Animals, Faculty of Veterinary Medicine, Ghent University, Salisburylaan 133, B-9820 Merelbeke, Belgium

**Keywords:** Proteoglycan, Growth retardation, Hypermobile joints, Galactosyltransferase I, Linkeropathy, Genome, Equus caballus, Extracellular matrix

## Abstract

**Background:**

Inbreeding and population bottlenecks in the ancestry of Friesian horses has led to health issues such as dwarfism. The limbs of dwarfs are short and the ribs are protruding inwards at the costochondral junction, while the head and back appear normal. A striking feature of the condition is the flexor tendon laxity that leads to hyperextension of the fetlock joints. The growth plates of dwarfs display disorganized and thickened chondrocyte columns. The aim of this study was to identify the gene defect that causes the recessively inherited trait in Friesian horses to understand the disease process at the molecular level.

**Results:**

We have localized the genetic cause of the dwarfism phenotype by a genome wide approach to a 3 Mb region on the p-arm of equine chromosome 14. The DNA of two dwarfs and one control Friesian horse was sequenced completely and we identified the missense mutation ECA14:g.4535550C > T that cosegregated with the phenotype in all Friesians analyzed. The mutation leads to the amino acid substitution p.(Arg17Lys) of xylosylprotein beta 1,4-galactosyltransferase 7 encoded by *B4GALT7*. The protein is one of the enzymes that synthesize the tetrasaccharide linker between protein and glycosaminoglycan moieties of proteoglycans of the extracellular matrix. The mutation not only affects a conserved arginine codon but also the last nucleotide of the first exon of the gene and we show that it impedes splicing of the primary transcript in cultured fibroblasts from a heterozygous horse. As a result, the level of *B4GALT7* mRNA in fibroblasts from a dwarf is only 2 % compared to normal levels. Mutations in *B4GALT7* in humans are associated with Ehlers-Danlos syndrome progeroid type 1 and Larsen of Reunion Island syndrome. Growth retardation and ligamentous laxity are common manifestations of these syndromes.

**Conclusions:**

We suggest that the identified mutation of equine *B4GALT7* leads to the typical dwarfism phenotype in Friesian horses due to deficient splicing of transcripts of the gene. The mutated gene implicates the extracellular matrix in the regular organization of chrondrocyte columns of the growth plate. Conservation of individual amino acids may not be necessary at the protein level but instead may reflect underlying conservation of nucleotide sequence that are required for efficient splicing.

**Electronic supplementary material:**

The online version of this article (doi:10.1186/s12864-016-3186-0) contains supplementary material, which is available to authorized users.

## Background

**Fig. 1:**
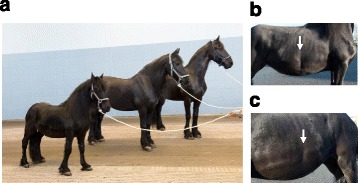
Dwarfism in the Friesian horse breed. **a** A female dwarf next to two normal female Friesian horses. The dwarf has a height at the withers of 1.12 m; the horse in the middle has a height of 1.54 m, which is close to the minimum allowed by the breed standard (1.53 m); the horse on the right has a height of 1.66 m. **b** and **c** Photographic illustrations of the typical pectus excavatum phenotype in the Friesian dwarf of the right **b** and the left side **c**

A dwarfism trait has been segregating in the Friesian horse breed for decades [1], (OMIA 000299-9796 [2]). Characteristic for the trait is the physeal growth retardation of limbs and ribs, resulting in a disproportionate form of dwarfism. The affected horses exhibit hyperextension of the fetlock joints of all limbs with varying severity. Flexor tendon laxity, which is often seen in newborn foals of all breeds, fails to recover in dwarf foals and instead tends to increase further during aging. As a consequence, these dwarf Friesians develop an abnormal gait in which the limbs undergo extreme outward rotation at the level of carpus and hocks. The ribcage is abnormal in most cases with thickened and S-shaped costochondral junctions, leading to an inward protrusion of the chest at the level of Th10-16 (Fig. 1b,c). Mature dwarfs have a head of the same size as unaffected horses, a broader chest with narrowing at the costochondral junction, a disproportionally long back and abnormally short limbs. The abdomen has a weak and rounded appearance, and the musculature over the body is poorly developed. Involvement of the hypothalamic-pituitary growth axis in the pathogenesis of the condition has been excluded [3].

A monogenic recessive mode of inheritance is most likely and, considering the breed structure with a small number of founders and narrow population bottlenecks, it is expected that the dwarfs are homozygous for the responsible gene mutation [4]. A genome wide association study of 10 cases and 10 control Friesian horses has been reported earlier [4]. The dwarfism locus was assigned to the telomeric region of the p-arm of chromosome 14 (ECA14), although genome wide significance was not reached. The aim of the present study was to confirm and further define the critical chromosome region of the locus and to identify the responsible gene mutation. The identification of the gene enables the comparison of the phenotype across species and enhances the understanding of the processes of growth and development.

## Results

### Gene mapping

**Fig. 2:**
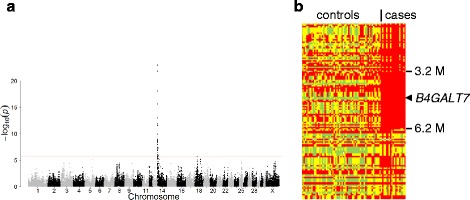
Localization of the dwarfism locus in Friesian horses. **a** Manhattan plot of the genome wide association study. The DNA of 19 dwarfs and 65 controls of the Friesian horse breed was typed with the Illumina EquineSNP50 array. The allele frequency differences between the groups were assessed with GenABEL software and plotted as the –log_10_p value from Chi-square tests. The red line indicates the Bonferroni corrected significance level. **b** Homozygosity mapping. Individual genotypes of informative SNPs from the telomeric region (0 - 10 Mb) of ECA14 of cases and controls were extracted from the array data. The results of the telomeric 134 SNPs of the p-arm that passed quality control are shown. Red: homozygous genotype of major allele in the dwarfs; green: homozygous genotype of minor allele; yellow: heterozygous genotype. The region of homozygosity in the dwarfs is bordered by the SNPs BIEC2-239119 and BIEC2-240544

To substantiate the localization of the dwarfism gene on chromosome ECA14 we performed a genome wide comparison of a group of dwarfs with a group of controls from the Friesian horse breed. The allelic association reached genome wide significance in the telomeric region of the p-arm of ECA14 with a Bonferroni corrected Pgenome = 2.90 × 10^−19^ for BIEC2-239391 at position 3776009, the SNP that was most significantly associated with dwarfism (Fig. 2a). In total 35 SNPs passed the Bonferroni corrected significance level (1.68 × 10^−6^) and all were located on ECA14 between positions 1 and 9510581. Inspection of the genotypes of the individual horses in the region showed that only the dwarfs shared a haplotype of 3 Mb homozygously, confirming that the phenotype originated from a single founder (Fig. 2b). The genotypes clearly pointed to recombination events that were evident in several cases and that placed the critical region between positions 3151847 and 6229282 on ECA14. According to the annotation release 101 of the NCBI of the equine reference genome the critical region contained 66 genes [5].

### DNA sequence analysis

**Fig. 3:**
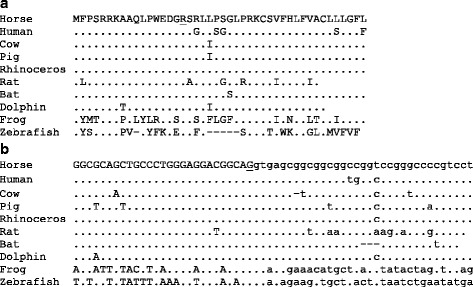
Conservation of *B4GALT7*. **a** Alignment of the N-terminal amino acid sequence of xylosylprotein beta 1,4-galactosyltransferase, polypeptide 7 of horse with that of several mammals, an amphibian and a fish. The protein sequences are derived from the reference cDNAs listed below. **b** Alignment of the splice donor site of exon 1 of *B4GALT7* of horse with that of other vertebrate species. The exon DNA sequence is in upper case and that of the intron is in lower case. The residues in **a** and **b** that are mutated in Friesian horse dwarfs are underlined. Residues that are identical to those in horse are indicated by a dot; gaps that are introduced to optimize the alignment are indicated by a dash. The exon 1/intron 1 splice junctions are derived from the respective reference genomes and based on the following cDNA reference sequences. Horse (Equus caballus): XM_014730464; human: NM_007255; cow (Bos taurus): NM_001075321; pig (Sus scrofa): NM_001168422; rhinoceros (Ceratotherium simum): XM_010145472; rat (Rattus norvegicus): NM_001031661; bat (Eptesicus fuscus): XM_008143179; Dolphin (Tursiops truncatus): XM_004313659; frog [Xenopus (Silurana) tropicalis]: (NM_001126545); zebrafish (Danio rerio): NM_001003417

Full genome DNA sequence data was generated of four dwarfs and three control Friesian horses by Next Generation Sequencing. The DNA sequence of the critical region of ECA14 of the dwarfs was compared with those of the controls, the reference genome, and the Quarter Horse that is publicly available [6]. As dwarfism has not been reported in the Quarter Horse breed we assumed that the causative mutation is not present in this population and that the horse was homozygous for the reference allele. The variations of the dwarfs as compared with the reference genome were filtered by snpSift [7] for possible effects on amino acid sequence or splicing (Additional file 1). Using the Integrative Genomics Viewer [8] we then searched for the variations that were absent in the Quarter Horse and not homozygously present in the control Friesian horses. Only one nonsynonymous mutation fulfilled these criteria. The mutation was ECA14:g.4535550C > T in *B4GALT7* and corresponds to XM_014730464.1:c.50G > A and XP_014585950.1:p.(Arg17Lys). Arginine and lysine are both basic amino acids that are interchangeably seen in many conserved protein domains. In this case, however, the arginine residue at position 17 of the equine *B4GALT7* encoded protein xylosylprotein beta 1,4-galactosyltransferase, polypeptide 7 (galactosyltransferase I) is strictly conserved in all vertebrates analyzed (Fig. 3a). Nonetheless, the mutation was considered moderate by the snpSift analysis and benign by PolyPhen-2 [9].

**Fig. 4:**
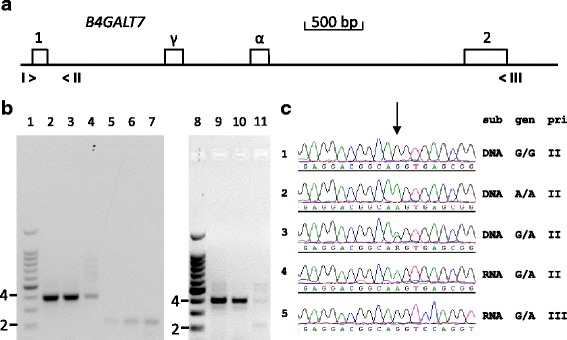
Mutation of the terminal nucleotide of exon 1 of *B4GALT7* affects proper RNA splicing of intron 1. **a** Map of the exon 1- exon 2 region of the equine *B4GALT7* gene. The positions of the exons 1 and 2 are indicated by numbered boxes; α: alternative exon; γ: cryptic exon. The position of the used PCR primers are indicated by > and < signs with Roman numerals. I>: EX1F; <II: IN1R; <III: EX2R. **b** cDNA fragments derived from a Friesian horse homozygous for the reference allele (lanes 2, 5 and 9), a heterozygous carrier (lanes 3. 6 and 10) and a Friesian horse dwarf (lanes 4, 7 and 11). Fibroblasts were grown from skin biopsies from the horses and RNA was isolated. cDNA was synthesized with reverse transcriptase followed by PCR with equine *B4GALT7* specific primers. Lanes 1 and 8: 100 bp size standard ladder (2 = 200 bp fragment, 4 = 400 bp fragment); lanes 2-4: primers I and II; lanes 5-7: primers I and III; lanes 9-11: primers I, II and III. **c** Genomic DNA and RNA sequence analysis of *B4GALT7* fragments from a horse homozygous for the reference allele (G/G), a dwarf (A/A) and a heterozygous carrier (G/A). The arrow indicates the position of the mutation. Sub: substrate genomic DNA (DNA) from the horses representing the three genotypes (gen) or cDNA synthesized with RNA from fibroblasts of the heterozygous horse (RNA). Pri: The PCR fragments were generated with primer I and either primer II or III as indicated. The mutant allele A is only observed in the unspliced RNA fragment (line 4) while the properly spliced product from the same heterozygous horse only shows the reference allele G (line 5)

The association of the mutation with the dwarfism phenotype was evaluated by Sanger DNA sequencing. All 29 dwarfs of which DNA was available were homozygous for the mutation (Fig. 4c, line 2). The 8 obligate carriers were heterozygous (line 3) and of a group of 177 Friesian horses 22 were carrier of the mutation and 155 were homozygous for the reference allele (line 1).

### RNA analysis

The mutated guanosine nucleotide is the last residue of exon 1 of *B4GALT7* and the position of this first splice donor relative to the start codon of the gene is conserved in vertebrates (Fig. 3b). The nucleotide is second in the triplet coding for arginine and since this amino acid is conserved, the guanosine is conserved with it. We wondered whether the selection pressure could have worked the other way around; that is, that the nucleotide itself was conserved not due to its amino acid coding properties but due to its splicing function. The mutation of guanosine to adenosine might affect splicing of the primary RNA transcript of the gene and splicing requirements may block its propagation. According to the splice site predictor NNSPLICE 0.9 [10] the exon 1/intron 1 junction of the equine reference gene had a splice donor score of 0.96 on a scale of 0 to 1. The mutated nucleotide sequence of the dwarfs had a moderate score of 0.58, suggesting that the mutation could indeed interfere with splicing.

To investigate the effect of the mutation on splicing of the primary transcript we isolated RNA from cultured skin fibroblasts of a Friesian horse dwarf, of a heterozygous carrier of the *B4GALT7* mutation and of a Friesian horse that was homozygous for the reference allele. Synthesis of cDNA was followed by PCR with a forward primer derived from *B4GALT7* exon 1 and a reverse primer from intron 1 or exon 2 (Fig. 4a). The RNA of the wildtype horse and the heterozygous horse yielded a splicing product with the exonic primers of the expected length of 401 bp (Fig. 4b, lanes 2 and 3). This product was less pronounced in the cDNA from the dwarf (lane 4). The primer set that included the intron 1 reverse primer produced a cDNA band derived from unspliced RNA of approximately 220 bp. This band was detected with the samples from the heterozygous horse and from the dwarf (lanes 6 and 7), but also, be it weakly, with the sample from the wildtype horse (lane 5). Combination of the 3 primers in a semi quantitative PCR showed spliced and unspliced products with similar intensities in the cDNA from the dwarf (lane 11). The wild type horse and the heterozygous horse do not display the unspliced product of 220 bp with the 3 primer PCR (lanes 9 and 10).

When we analyzed the cDNA sequence of the unspliced product from the heterozygous horse (lane 6) it was derived from the mutant allele only (Fig. 4c, line 4). The cDNA sequence of the properly spliced product from the same horse (lane 3) indicated that it was derived from the normal allele (Fig. 4c, line 5). The *B4GALT7* cDNA analysis confirms that the mutation r.50 g > a leads to a splicing deficiency of the primary transcript.

The exon 1/exon 2 primer set produced minor fragments that were larger than the expected length (Fig. 4b, lane 4). DNA sequence analysis of the fragments derived from the dwarf showed that the fragment of approximately 560 bp contained an alternative exon of 162 nucleotides between exon 1 and exon 2 sequences that were spliced at the proper positions. This alternative exon is annotated as such in Genbank. It is situated from position 4533657 to 4533818 of chromosome 14 of the reference genome EquCab 2.0. It should be noted that the gene is situated in the reverse orientation on the chromosome. The alternative exon has an open reading frame that is in frame with the remainder of the coding sequence of the gene and the amino acid sequence encoded by the exon is conserved in mammals.

Another minor cDNA fragment of approximately 730 bp contained the same alternative exon sequence together with a cryptic exon of 169 nucleotides located between exon 1 and the alternative exon. The genomic location of the cryptic exon was from 4534375 to 4534543 on chromosome 14 and it contained multiple stopcodons in frame with the start codon on exon 1.

The aberrant splicing from the exon 1 donor due to the variant r.50 g > a was associated with a severe reduction of expression of *B4GALT7* at the mRNA level. Quantitative PCR measurement of cDNA fragments indicated that the concentration of transcripts from the gene in the fibroblasts from the dwarf was only 2 % of that in fibroblasts from a Friesian horse that did not carry the mutation (Additional file 2).

## Discussion

Disproportionate dwarfism in Friesian horses is associated with a mutation in *B4GALT7*. The mutation changes a conserved arginine codon to a lysine codon. Both amino acid residues are basic and the effect of the mutation is considered moderate by the snpSift prediction. The mutation also affects the last nucleotide of exon 1 of the gene. Unspliced cDNA fragments spanning the exon 1/intron 1 junction can be detected regardless of the genotype of the horses. However, the cDNA sequences from a heterozygous horse clearly show that RNA derived from the mutant allele is hardly spliced, in contrast to the RNA from the normal allele (Fig. 4c, lines 4 and 5).

When an exonic and an intronic reverse primer are allowed to compete in a 3 primer PCR, only the cDNA from the dwarf displays the spliced and unspliced products in comparable amounts (Fig. 4b, lane 11). The normally spliced product is seen prominently in the wild type horse and the horse heterozygous for the mutation, but the unspliced product cannot be discerned among the products from these horses (lanes 9 and 10). This semi quantitative PCR and the cDNA sequence analysis of the products of the heterozygous horse confirms that the *B4GALT7* mutation strongly reduces the splicing capacity of the exon 1/intron 1 junction.

In the homozygous state, the mutation leads to low mRNA levels and the expression of the gene is strongly reduced as measured by qPCR. The improperly spliced transcripts could be prone to nonsense mediated decay.

The nucleotide sequence AGgt of the exon 1 splice junction of *B4GALT7* and its position with regard to the start codon are highly conserved (Fig. 3b). One could argue that the last nucleotide of the exon is expected to be conserved if the encoded arginine residue would be essential for the function of the protein. This G residue is the second nucleotide of the codon and all six triplets that code for arginine have a G residue at the second position. Thus, if the arginine is conserved, the guanosine is conserved with it. The first position of the codon under consideration is a conserved A residue, while 4 of the 6 possible arginine codons start with a C. A functional restriction on the encoded arginine residue would therefore not necessarily lead to conservation of the A residue of the AGgt splice junction. The hypothetical mutation of the A residue to a C would only lead to a moderate drop of the splice donor score from 0.96 to 0.89. According to this prediction a mutation of the second to last A residue to a C would be allowed while in fact it is highly conserved. Recently, a mutation of the A residue of a splice donor site in *IBA57* with the same AGgt junction sequence as exon 1 of *B4GALT7* was shown to impede proper splicing, causing a severe leukoencephalopathy [11]. This mutation did not alter the encoded amino acid and it stresses the importance of the exonic terminal nucleotide sequences for splicing at particular junctions. An *in vitro* splicing assay may resolve the importance of the second to last A residue of the exon 1 of *B4GALT7* for proper splicing. Concurrent with our results, the NNSPLICE program assigns a much lower splice donor score of 0.58 for the mutation found in the Friesian dwarfs. Considering all our results, we conclude that the conservation of the exon 1 terminal sequence in vertebrates reflects a restriction on a splicing requirement rather than on a functional requirement of the encoded amino acid. Characterization of naturally occurring mutations that are uncovered because of an association with disease can render important insights in splicing requirements [12].

In humans, mutations in *B4GALT7* cause the Ehlers-Danlos syndrome, progeroid type 1 (EDSP1, OMIM130070) and Larsen of Reunion Island syndrome (LRS). Only 7 mutations have been described in relation to the recessively inherited syndromes [13–18]. Most patients were normal with respect to length and weight at birth but soon were presented with growth retardation, osteopenia, facial dysmorphology, loose joints, bone dysplasias, loose skin and in most cases mild forms of mental retardation. Pectus carinatum was reported for a number of patients [16, 17]. The human phenotype is highly variable, even in patients sharing the same mutation homozygously [17].

A founder effect in a closed population of Reunion has led to at least 22 cases of LRS that were genetically confirmed. LRS was described as a subtype of Larsen syndrome [19]. The same mutation that causes LRS was observed homozygously in two siblings from another population diagnosed with EDSP1. The progeroid aspect was not observed in any of the genetically confirmed cases of EDSP1 nor LRS and it has been suggested to remove this term from the name of the EDSP1 syndrome [16, 18].

Clear similarities between the conditions in man and horse are growth retardation and hypermobile joints. Rib deformities have been observed in human as well as equine cases [1, 16, 17]. Pectus carinatum, reported in human cases, refers to the pectus in which the ribcartilage has been overdeveloped outward, leading to a ‘chicken chest’. In the Friesian horse cases on the other hand, the ribcartilage has been overdeveloped inward, leading to pectus excavatum or ‘shoemaker chest’ in humans.

The dwarfism in the horse is described as a disproportionate growth disturbance because all limbs are short, while the head and back appear rather normal. In contrast, almost all confirmed human patients with LRS and EDSP1 display facial dysmorphism and disproportional growth was not noted [16, 17]. Cognitive functions do not seem to be impaired in dwarf horses. Another clear difference between the phenotype in man and horse is that loose skin has never been observed in Friesian dwarfs. Atrophic scarring and/or delayed wound healing has been reported for a number of human patients but is never seen in Friesian horse dwarfs. The fibroblasts from one human patient displayed reduced proliferation rates [20], while the fibroblasts of the Friesian dwarf proliferated at least as fast as the fibroblasts from normal Friesians. The differences in the clinical presentations between human patients and Friesian dwarfs may be due to the nature of the mutation in horses, which we think has predominantly an effect on the expression level of a normally functioning protein. On the other hand, the protein may have rate limiting key roles in processes that are different in the two species, such that loss of activity becomes manifest in different ways.

The *B4GALT7* gene is highly expressed in the proliferative zone of the growth plate in rat [16]. Deficiency of the encoded xylosylprotein beta-1,4-galactosyltransferase 7 apparently induces the irregularities of the chondrocyte columns seen in the growth plate of Friesian dwarfs [1]. The enzyme adds the second of four saccharides that form the linker between the protein core and the glycosaminoglycan moiety of proteoglycans. Proteoglycans are major components of molecular networks of the extracellular matrix. Mutations in any of the enzymes that build the saccharide linker cause a variety of rare syndromes with overlapping features, which are called linkeropathies (reviewed in [21]). Dwarfism in Friesian horses could therefore be considered as a new presentation of a linkeropathy.

Remarkably, this is the second gene with a role in protein glycosylation in which we found a pathogenic mutation in Friesian horses. Earlier we found a nonsense mutation in *B3GALNT2* involved in muscular dystrophy with hydrocephalus in stillborn foals [22]. The encoded beta-1,3-N-acetylgalactosaminyltransferase is involved in glycosylation of alpha-dystroglycan, which is part of the complex that connects the cytoskeleton with the extracellular matrix.

## Conclusions

We provide evidence indicating that dwarfism in Friesian horses could be caused by a splicing deficiency of *B4GALT7* that severely reduces expression of the gene. The conservation of the affected nucleotide reflects a splicing requirement rather than a functional requirement of the encoded amino acid. The clinical picture of the Friesian horse dwarfs adds to the phenotypic variability observed in human patients with *B4GALT7* mutations. Crosses between carriers can be prevented by screening breeding horses for the *B4GALT7* mutation and the dwarfism trait could thus be eliminated from the breed.

## Methods

### Phenotypes, genotypes and genome-wide association study

Friesian horses (*n* = 29) were diagnosed as being dwarfs by local equine veterinarians in consultation with the Equine Clinic of Utrecht University, usually via a digital *in vivo* picture for confirmation of the phenotype. Thirteen of the horses were male, 11 female and the sex of 5 dwarfs was unknown. The group of unaffected controls (*n* = 65) were Friesian horses without the characteristic appearance of dwarfism [1]. In addition, we obtained blood samples for DNA isolation from 8 parents of dwarfs and DNA samples from 177 Friesian horses that were part of a DNA bank maintained at the Dr. Van Haeringen Laboratory B.V..

Blood samples were taken and DNA was isolated as described by Orr et al. [4]. Genotypes of 19 dwarfs and 65 controls were obtained using the Illumina® EquineSNP50 Genotyping BeadChip containing 54,602 SNPs. Quality control was performed using the check.marker function in the GenABEL package in R [23]. SNPs with MAF <5 % and call-rate <90 % were discarded, leaving 29,840 SNPs (54.7 % of all SNPs) for the analysis.

The ccfast function in GenABEL package in R [23] was used to determine the significance of allelic differences between dwarfs and unaffected horses with a χ^2^-test (1df). The Bonferroni corrected significance level applied was 1.68 × 10^−6^. Homozygosity mapping in the significantly associated region was performed by eye to identify overlapping regions of homozygosity between dwarfs.

### Genome sequencing

Four dwarf cases and three unrelated control were paired-end sequenced with 150 nucleotide reads for the full genome on Illumina NextSeq500 to an average coverage of 4-9x according to the manufacturers protocols. To increase the power to detect causal candidate variants as a fully homozygous variant, we merged the data for the four cases yielding an mean coverage of 36x.

The Illumina data was processed with our inhouse developed pipeline v 1.2.1 [24] including GATK v3.2.2 [25] according to the best practices guidelines [26]. Briefly, we mapped the pairs with BWA-MEM v0.7.5a [27], marked duplicates, merged lanes, realigned indels. Base recalibration did not improve our results, so this step was skipped. Next, GATK Haplotypecaller was used to call SNPs and indels. Variants are flagged as PASS only if they do not meet the following criteria: QD < 2.0, MQ < 40.0,FS > 60.0, HaplotypeScore > 13.0, MQRankSum < -12.5, ReadPosRankSum < -8.0, snpclusters > =3 in 35 bp. For indels: QD < 2.0, FS >200.0, ReadPosRankSum < -20.0.

Detecting recessive candidate variants was done with snpSift [28] fitting the model of reference or potential carrier status in the control and homozygous state in the cases. Moreover, coverage > 10, a genotypequality of >30 and effect impact ‘HIGH’ or ‘MODERATE’ was required. Additional evaluation of the variant of interest was performed with PolyPhen-2 [9].

The observed possibly detrimental DNA variant of *B4GALT7* was confirmed and evaluated in the complete cohort by Sanger sequencing of PCR fragments. The PCR primer sequences were 5’- AGTTTCTCGGAGTGTAGAG-3’ (UP1F) and 5’-AGAGACATAGACCCTCAGAG-3’ (IN1R). The PCR was performed with 50 ng genomic DNA, 3 U Platinum Taq DNA polymerase (Thermo Fisher Scientific, Waltham, MA), 2 mM MgCl2, 0.2 mM each dNTP, 0.5 μM each primer, 1 M betaine and 1× Platinum buffer. Temperature cycling conditions were 5 min at 95 °C, 35 cycles of 30 s at 95 °C, 30 s at 55 °C, 30 s at 72 °C, and a final elongation step at 72 °C for 10 min. All amplifications were performed on an ABI 9700 Thermal Cycler (Applied Biosystems, Foster City, CA). The PRC primers were degraded by addition of 1 U Exonuclease I (Thermo Fisher Scientific, Waltham, MA) and incubation for 15 min. at 37 ^o^C and 15 min. at 85 ^o^C. DNA sequencing tercycle reactions were performed using BigDye v3.1 (Thermo Fisher Scientific, Waltham, MA) according to the manufacturer’s protocol. The products were analysed on a 3130XL Genetic Analyzer (Applied Biosystems, Foster City, CA) and the data was analysed with Lasergene (version 11 DNASTAR).

Homologous DNA and protein sequences from different species were retrieved from GenBank and aligned one by one by eye. Identities and differences were indicated by using a word processor. The species were selected arbitrary to represent close and distant members of the animal kingdom.

### RNA analysis

Fibroblasts were grown from 6 mm punch biobsies from the skin of a dwarf, a carrier of the mutation of interest and a Friesian horse homozygous for the reference allele. The biopsies were washed in Euroflush (IMV technologies, L’Aigle, France) containing 5000 IU/ml heparin, cut with scissors and incubated in petri dishes with DMEM/M199 (1:1) medium containing pen/strep (10,000 U/ml (all from Thermo Fisher Scientific, Waltham, MA), 2.5 ng/ml basic-FGF5 (Peprotech, Rocky Hill, NJ) and 20 % FCS at 38.5 ^o^C with 5 % CO_2_ and 5 % O_2_. Proliferating fibroblasts were harvested and passaged in culture flasks using standard procedures. RNA was isolated from cultured fibroblasts with the RNeasy kit with an on-column DNase digestion according to the instructions of the manufacturer (Qiagen, Hilden, Germany). The RIN value of the RNA was meausured with an Agilent 2100 Bioanalyzer (Santa Clara, CA, USA) and was found to be 9.5 or higher. cDNA was synthesized with the iScript kit (Bio-Rad Laboratories, Hercules, CA) using 500 ng of RNA in 20 μl reactions. Splicing products were PCR amplified from 0.5 μl cDNA product with *B4GALT7* exonic primers 5’-CTGGGAGCTCGAGCTCCATG-3’ (EX1F) and 5’-CTCAGGAAGCGGTGCATGTG-3’ (EX2R) as described above. Unspliced products were amplified with primer EX1F and the intronic primer IN1R described above. In a semi quantitative experiment, the 3 primers were combined in a single PCR using the same component concentrations and cycling conditions as above. The fragments were visualized by electrophoresis on a 1 % agarose gel in 0.5x TBE with 0.5 μg/ml ethidium bromide followed by UV irradiation of the gel.

For DNA sequence analysis and confirmation of the origin of the products, the bands were cut from the gel and the DNA was isolated with QIAquick gel extraction kit (Qiagen, Hilden, Germany). The DNA sequencing procedure was as described above.

Quantitative PCR for measurement of *B4GALT7* transcripts was performed with forward primer 5’- GACGGCAGGTCCAGGTTG-3’ and reverse primer 5’- ACAGGCAACGAAGAGGTGG-3’ at an annealing temperature of 55 ^o^C. The forward primer bridges exons 1 and 2, while the reverse primer is situated in exon 2. The qPCR reactions contained 1x IQ SYBRGreen SuperMix (Bio-Rad, Laboratories, Hercules, CA), 400 nM of each primer and 1 μl cDNA, obtained as described above, in a total volume of 15 μl. The reference genes were *RPS19* and *RPL13A*, which were analyzed as described [29]. The reactions were performed in a MyiQ2 thermal cycler and the data was analyzed with IQ5 software (both from Bio-Rad, Laboratories, Hercules, CA).

## Additional files

Additional file 1: Variants with predicted moderate or high effect on gene function in the critical chromosome region of Friesian dwarf horses. Output of snpSift analysis of Next Generation Sequence data. (XLSX 15 kb)
Additional file 2: Expression of *B4GALT7* in relation to genotype. qPCR data of *B4GALT7* mRNA from fibroblasts. (XLSX 13 kb)

## Abbreviations

ECA14: Equus Caballus chromosome 14
EDSP1: Ehlers-Danlos syndrome, progeroid type 1
KFPS: Koninklijke Vereniging “Het Friesch Paarden-Stamboek” (i.e. Royal Friesian Horse Studbook)
LRS: Larsen of Reunion Island syndrome.
